# Identification of a 251 Gene Expression Signature That Can Accurately Detect *M. tuberculosis* in Patients with and without HIV Co-Infection

**DOI:** 10.1371/journal.pone.0089925

**Published:** 2014-02-25

**Authors:** Noor Dawany, Louise C. Showe, Andrew V. Kossenkov, Celia Chang, Prudence Ive, Francesca Conradie, Wendy Stevens, Ian Sanne, Livio Azzoni, Luis J. Montaner

**Affiliations:** 1 Center for Systems and Computational Biology, The Wistar Institute, Philadelphia, Pennsylvania, United States of America; 2 Genomics Facility, The Wistar Institute, Philadelphia, Pennsylvania, United States of America; 3 Clinical HIV Research Unit, Department of Clinical Medicine, Faculty of Health Sciences, University of the Witwatersrand, Johannesburg, South Africa; 4 Department of Molecular Medicine and Hematology, Faculty of Health Sciences, University of the Witwatersrand, Johannesburg, South Africa; 5 HIV-1 Immunopathogenesis Laboratory, The Wistar Institute, Philadelphia, Pennsylvania, United States of America; The Ohio State University, United States of America

## Abstract

**Background:**

Co-infection with tuberculosis (TB) is the leading cause of death in HIV-infected individuals. However, diagnosis of TB, especially in the presence of an HIV co-infection, can be limiting due to the high inaccuracy associated with the use of conventional diagnostic methods. Here we report a gene signature that can identify a tuberculosis infection in patients co-infected with HIV as well as in the absence of HIV.

**Methods:**

We analyzed global gene expression data from peripheral blood mononuclear cell (PBMC) samples of patients that were either mono-infected with HIV or co-infected with HIV/TB and used support vector machines to identify a gene signature that can distinguish between the two classes. We then validated our results using publically available gene expression data from patients mono-infected with TB.

**Results:**

Our analysis successfully identified a 251-gene signature that accurately distinguishes patients co-infected with HIV/TB from those infected with HIV only, with an overall accuracy of 81.4% (sensitivity = 76.2%, specificity = 86.4%). Furthermore, we show that our 251-gene signature can also accurately distinguish patients with active TB in the absence of an HIV infection from both patients with a latent TB infection and healthy controls (88.9–94.7% accuracy; 69.2–90% sensitivity and 90.3–100% specificity). We also demonstrate that the expression levels of the 251-gene signature diminish as a correlate of the length of TB treatment.

**Conclusions:**

A 251-gene signature is described to (a) detect TB in the presence or absence of an HIV co-infection, and (b) assess response to treatment following anti-TB therapy.

## Introduction

*Mycobacterium tuberculosis* (MTB) (tuberculosis, TB) is the leading infectious disease cause of mortality and morbidity worldwide [1]. An estimated 30% of the world population is infected with TB [2]. The concurring epidemic of HIV infection in areas endemic for TB infection has resulted in a high burden of HIV/TB co-infection, and TB is currently estimated to be the leading cause of death in HIV-infected patients in these areas [3], [4], [5], [6]. The weakening of the host’s immune system by HIV increases risk of de-novo co-infection with TB, or reactivation of latent TB [7], [8], [9]. The emergence of multidrug-resistant and extensively drug-resistant (MDR and XDR) TB strains has further taxed the healthcare systems in high-TB burden countries. The presence of an HIV infection has been associated with, and may contribute to, the increase in MDR-TB cases [6], [10], highlighting the importance of research into novel treatments and new diagnostic tools for early detection of TB infections.

Current diagnostic methods are associated with several limitations including sample collection issues associated with the automated sputum based diagnostic test that can identify MTB and resistance to rifampicin (Xpert MTB/RIF) [11]) or limited sensitivity and specificity associated with testing for urinary lipoarabinomannan (LAM) [12], [13], [14], [15]). In addition, while LAM detection methods have greater sensitivity in advanced disease [16], even a combination of Xpert MTB/RIF and LAM tests can only detect approximately 80% of symptomatic patients [17]. The presence of an HIV infection further limits the sensitivity for the diagnosis of TB as it increases the number of false negatives detected [18], [19]. Failure to detect TB early in HIV co-infected patients is lethal, and better assays for diagnosing TB are needed to reduce the high level of mortality caused by TB in these patients [18].

The interaction of TB with the host immune system induces changes in host gene expression. Ribonucleic acid (RNA)-based methods such as messenger RNA microarrays that interrogate the whole transcriptome have prompted efforts to detect specific host gene expression signatures correlated with different aspects of disease [20]. Several recently published gene expression studies have explored the biological mechanisms of TB infections and identified biomarkers that may be useful for diagnostic and prognostic purposes [21], [22], [23], [24], [25]. However, to our knowledge, no group has yet described a gene signature able to identify patients co-infected with HIV and TB. We address this problem in the present study by analyzing global gene expression in peripheral blood mononuclear cell (PBMC) samples from a South African cohort of 43 patients infected with HIV alone or co-infected with HIV and TB. Our analysis identifies a 251-gene signature that distinguishes mono-infected HIV patients from HIV patients co-infected with TB. The accuracy of this signature is 81.4% (sensitivity = 76.2%, specificity = 86.4%). This signature was validated on two large publicly available, independent gene expression datasets of patients infected with TB but not HIV, reported by Berry et al. [22] and Bloom et al. [26]. Our HIV/TB signature also accurately distinguished patients mono-infected with TB from healthy individuals and patients with latent TB. It also distinguished untreated infected patients from patients undergoing progressive to successful anti-TB treatment, suggesting the potential to monitor response to therapy.

## Methods

### Subjects

Study subjects were recruited between September 6, 2007 and October 16, 2008 at the Themba Lethu Clinic, Johannesburg, South Africa and included 22 HIV mono-infected patients and 21 HIV/TB co-infected patients (Table 1). Patients were referred to the Themba Lethu Clinic by the initial practitioners (HIV cohort) or primary TB clinic (HIV/TB cohort) in the Johannesburg catchment area for the initiation of antiretroviral therapy (ART) according to local guidelines. All patients were receiving ART at the time of enrollment and all patients co-infected with TB were receiving treatment for TB. Written informed consent was obtained for all participants; consent forms and procedures, as well as study protocol, were approved by the University of the Witwatersrand’s Ethics Committee and the Wistar Institute Institutional Review Board. Patients were initially recruited to study natural killer cell activity in HIV and HIV/TB infected patients and the results have been published by Conradie et al. [27]. PBMC were purified on location from peripheral blood using Ficoll gradient centrifugation, and cryopreserved, then shipped in a single batch to the Wistar Institute using a certified cold chain carrier in liquid nitrogen shippers. Samples remained in liquid nitrogen until the time of RNA extraction.

**Table 1:** Patient demographics.

	HIV	HIV/TB
Number of Patients	22	21
Age†	33.5 [31–38.75]	31 [30]–[35]
Gender: Male (Female)	6 (16)	10 (11)
Observed CD4 Counts at Time of Visit†	215 [127.75–339]	101 [78–180]
ART Treatments	3TC+D4T+EFV (21)	3TC+D4T+EFV (21)
	3TC+D4T+NVP (1)	
Length of ART Treatment(months)†	37 [28–56]	42 [28–56]

†Numbers are shown as median [first and third quartiles]. 3TC: Lamivudine, D4T: Stavudine, EFV: Efavirenz, and NVP: Nevirapine.

### RNA and Isolation, Amplification & Hybridization

Total RNA was isolated from PBMCs using Sigma Aldrich Tri-reagent (cat #T9424), as recommended, with the following modifications: 1 ug of linear acrylamide was added to the sample before Tri-reagent extraction to ensure more efficient precipitation of RNA and 1 ul of RNAsin (an RNAse inhibitor) was added to the Tri-reagent aqueous phase before continuing to the ethanol precipitation. Following RNA isolation, 100 ng of RNA was amplified using Epicentre TargetAmp Nano-g Biotin-aRNA Labeling Kit (cat # TAN07924) to generate amplified cRNA. Biotinylated, amplified cRNA at 750 ng was hybridized to the Illumina HumanHT-12 v4 Expression BeadChips. All arrays were processed in the Wistar Institute Genomics Facility. Gene expression data is available in the Gene Expression Omnibus (GEO) under the accession number GSE50834.

### Data Preprocessing

Raw gene expression microarray data were quantile normalized. Non-informative probes, which were either expressed at background level or showed little variation among samples such that the maximum fold change between any two samples was <1.2, were removed. Two technical replicates available for one patient were averaged prior to further analysis. Data preprocessing was performed in MATLAB R2010a.

### Support Vector Machines

The Support Vector Machine with Recursive Feature Elimination (SVM-RFE [28], [29], [30]) algorithm was implemented in Perl and used for the selection of features that can best distinguish mono-infected HIV patients from co-infected HIV/TB patients. The SVM classifier was iteratively trained with the current set of features and the least important features were then removed. The SVM-RFE parameters were set to 10 fold cross-validation with 10 iterations and 10% of the least informative features were eliminated at every step as previously described [29], [30]. SVM-RFE produced a ranked list of genes. A gene’s rank in the list correlates with its contribution to the overall TB signal, such that genes ranked at the top of the list contribute more to the predictive value of the classifier. Using the most predictive genes, the SVM classifier assigned a score to each of the samples in the training set, where a positive score indicates a prediction of TB and a negative score indicates a control (no active TB). The smallest number of genes that results in the highest classification accuracy (in this case 251 genes) makes up the TB signature or classifier. The sensitivity, specificity and accuracy of the classifier are evaluated at every recursive elimination step.

### Independent Validation Samples

The published external TB associated datasets GSE19435, GSE19439, GSE19442 and GSE19444 [22], available in the GEO database, were used to validate and expand our present study. In this published study [22], RNA from whole blood was hybridized to the Illumina HumanHT-12 v3 BeadChip Arrays. Since our study uses the more recent v4 arrays only the 13,880 probes that had passed the filtering criteria and were common to both Illumina’s v3 and v4 platforms were considered in any analyses. We normalized the data obtained from [22] by first calculating the average expression per probe across samples in each dataset separately and then across all samples from the 4 datasets. The difference between the average of a dataset and the overall average was determined and the expression level for each probe within a sample was adjusted by the difference. Data later obtained from GSE40553 [26] also using RNA from whole blood was first normalized using median quantile normalization. The data was then normalized with respect to the other 4 datasets by again adjusting each sample to the difference between the average expression for the dataset and the average across all 4 datasets. None of the samples used for validation were from HIV infected individuals.

Dataset GSE19435 [22] was used for feature selection and validation of our HIV/TB classifier. This dataset included 33 samples; 12 healthy controls and 7 TB patients with the TB samples taken before treatment, after 2 months of treatment and again after 12 months of treatment (7 samples for each time point, total 21). For the purpose of this analysis the dataset was divided into two test sets: Test Set 1 (TS1) containing data from TB patients after 2 months of treatment and healthy controls (Table 2), and Test Set 2 (TS2) containing data from TB patients before treatment and after 12 months of treatment (Table 2). TS1 was chosen to resemble the data used in our present study. TS2 was tested to confirm the presence of the HIV/TB signature in patients prior to treatment and to examine the expression of those genes after treatment.

**Table 2:** Test Set Summary.

			Controls		Active TB		
Dataset	Location	Test Set	Healthy	Latent TB	No Treatment	2 Months Treatment	12 Months Treatment
**GSE19435**	UK	TS1	12	–	–	7†	–
		TS2	–	–	7†	–	7†
**GSE19439**	UK	TS3	12	17	13	–	–
**GSE19444**	UK	TS4	12	21	21	–	–
**GSE19442**	South Africa	TS5	–	31	20	–	–
**GSE40553**	South Africa	TS6	–	38	29	–	–

Sample details of the six test sets.

†repeated sampling of the same patients.

The signature was further validated in four additional datasets from the same study [22]. Test Set 3 (TS3) contained data on 42 samples including 13 active TB patients, 17 latent TB patients and 12 controls. Test Set 4 (TS4) contained data on 21 active TB patients, 21 latent TB cases, and 12 controls. Test Set 5 (TS5) contained 51 samples, with data available from 20 active TB patients and 31 latent TB patients. And Test Set 6 (TS6) contained data on 29 active TB patients and 38 latent TB cases (Table 2). All controls and patients in Test Sets 1–6 were not infected with HIV [22], [26]. Each sample in the six training sets received an SVM score to indicate whether the classifier predicts the presence (positive score) or absence (negative score) of active TB.

## Results

### Identification of a 251-gene Signature that Accurately Distinguishes HIV/TB Co-infected from HIV Mono-infected Patients

**Figure 1:**
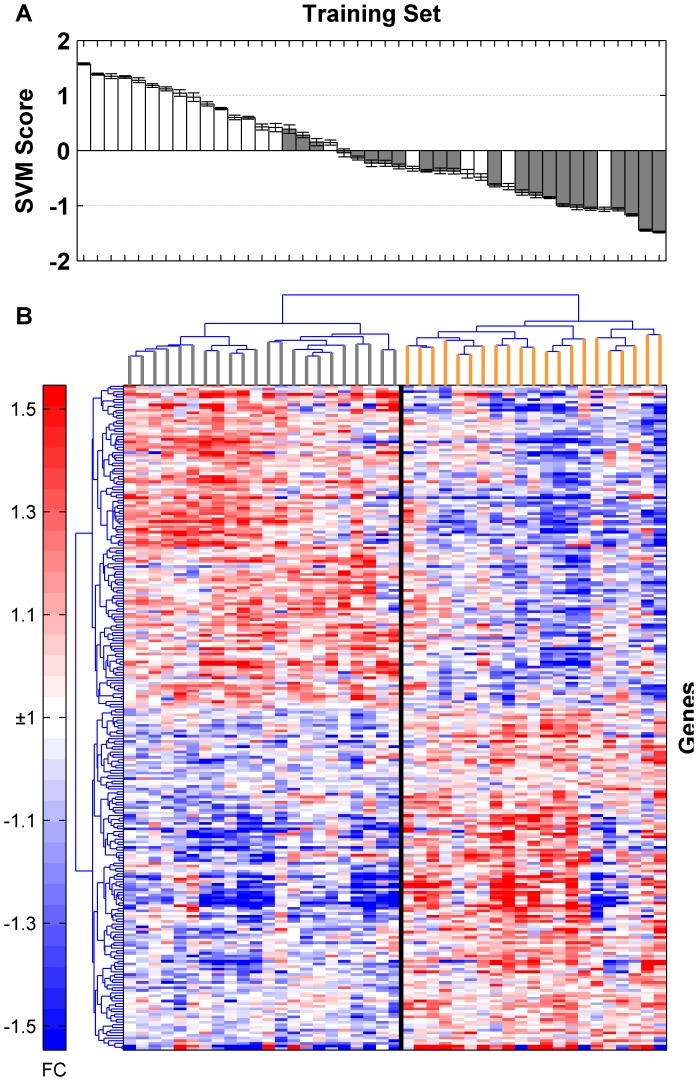
Classification of the training set samples. A) Support vector machine (SVM) scores based on the 251-gene signature assigned to HIV/TB (white) and HIV (grey) samples, where a positive score corresponds to an HIV/TB prediction and a negative score to a control, and B) Hierarchical clustering using the 251 probes clearly separate the samples into two arms, with mono-infected HIV patients (grey) clustering to the left and co-infected HIV/TB patients (orange) clustering to the right.

We analyzed global gene expression in PBMC derived from mono and co-infected HIV patients to identify a gene signature that could distinguish these two classes of patients. We identified a 251-gene signature that accurately distinguishes mono and co-infected patients (Table 3, Table S1) with an overall diagnostic accuracy of 81.4%. The 251 gene signature correctly classified 16 of the 21 HIV/TB patients (sensitivity = 76.2%) and 19 of the 22 HIV patients (specificity = 86.4%) (Table 4, Figure 1*A*). Hierarchical clustering analysis of the diagnostic gene signature was performed to further compare the differential expression between the two groups. The heatmap shows the separation of samples into two main clusters, with the left cluster including patients infected with HIV only and the right cluster representing HIV/TB co-infected patients (Figure 1*B*).

**Table 3:** Top 20 genes from the 251-gene TB-signature identified using SVM-RFE.

Rank	Accession	Symbol	Gene Name	Fold Change (HIV+TB)/HIV
1	NM_021004	DHRS4	dehydrogenase/reductase (SDR family) member 4	1.402
2	NM_001990	EYA3	eyes absent homolog 3 (Drosophila)	1.172
3	NM_030629	CMIP	c-Maf-inducing protein	1.248
4	NM_014233	UBTF	upstream binding transcription factor, RNA polymerase I	−1.260
5	NM_032227	TMEM164	transmembrane protein 164	1.492
6	NM_078487	CDKN2B	cyclin-dependent kinase inhibitor 2B (p15, inhibits CDK4)	1.422
7	NM_005607	PTK2	PTK2 protein tyrosine kinase 2	1.374
8	XM_939593	LOC648605	PREDICTED: similar to Trimethyllysine dioxygenase, mitochondrial precursor	1.256
9	CR621233	NaN	full-length cDNA clone CS0DI057YA22 of Placenta Cot25-normalized of (human)	1.441
10	NM_003501	ACOX3	acyl-Coenzyme A oxidase 3, pristanoyl	1.336
11	XM_001126647	MLKL	PREDICTED: mixed lineage kinase domain-like	1.454
12	NM_007100	ATP5I	ATP synthase, H+ transporting, mitochondrial F0 complex, subunit E	1.102
13	NM_005819	STX6	syntaxin 6	1.255
14	NM_152858	WTAP	Wilms tumor 1 associated protein	−1.586
15	NM_001531	MR1	major histocompatibility complex, class I-related	1.421
16	XM_045290	LOC151579	PREDICTED: similar to basic leucine zipper and W2 domains 1	−1.234
17	NM_025164	SIK3	SIK family kinase 3	1.131
18	XM_036729	USP41	PREDICTED: ubiquitin specific peptidase 41	1.253
19	NM_145172	WDR63	WD repeat domain 63	−3.149
20	NM_023015	INTS3	integrator complex subunit 3	1.275

Genes are listed according to the support vector machine (SVM) rank. A positive fold change indicates higher expression in HIV/TB compared to HIV and negative values indicate lower expression of the gene in HIV/TB compared to HIV. A full list of the 251 genes can be found in Table S1. SVM-RFE: support vector machine with recursive feature elimination. HIV: Human immunodeficiency virus. TB: Tuberculosis.

**Table 4:** Performance of classifier.

		Sensitivity		Specificity		Accuracy	AUC
Dataset	Description	SVM	KNN	SVM	KNN	SVM	SVM
HIV/TB	HIV/TB vs. HIV	76.2	NA	86.4	NA	81.4	0.864
TS1	TB (2 mo) vs. Controls	85.7	NR	100	NR	94.7	1.000
TS2	TB vs. TB (12 mo)	85.7	NR	100	NR	92.9	0.980
TS3	TB vs. Controls/Latent TB	69.2	91.7	100	96.6	90.5	0.936
TS4	TB vs. Controls/Latent TB	76.2	61.7	97.0	93.8	88.9	0.893
TS5	TB vs. Latent TB	90.0	94.1	90.3	96.7	90.2	0.924
TS6	TB vs. Latent TB	86.2	NA	92.1	NA	89.6	0.967

Sensitivity, specificity, accuracy and the area under the curve of the 251-gene signature for the training set (HIV/TB) and the 6 different test sets (SVM). Sensitivities and specificities reported in [22] using the K-nearest neighbor (KNN) method are also reported where applicable. AUC: Area under the curve. NA: not applicable and NR not reported.

### The 251-gene HIV/TB Signature also Accurately Detects Active TB in the Absence of HIV Infection

**Figure 2:**
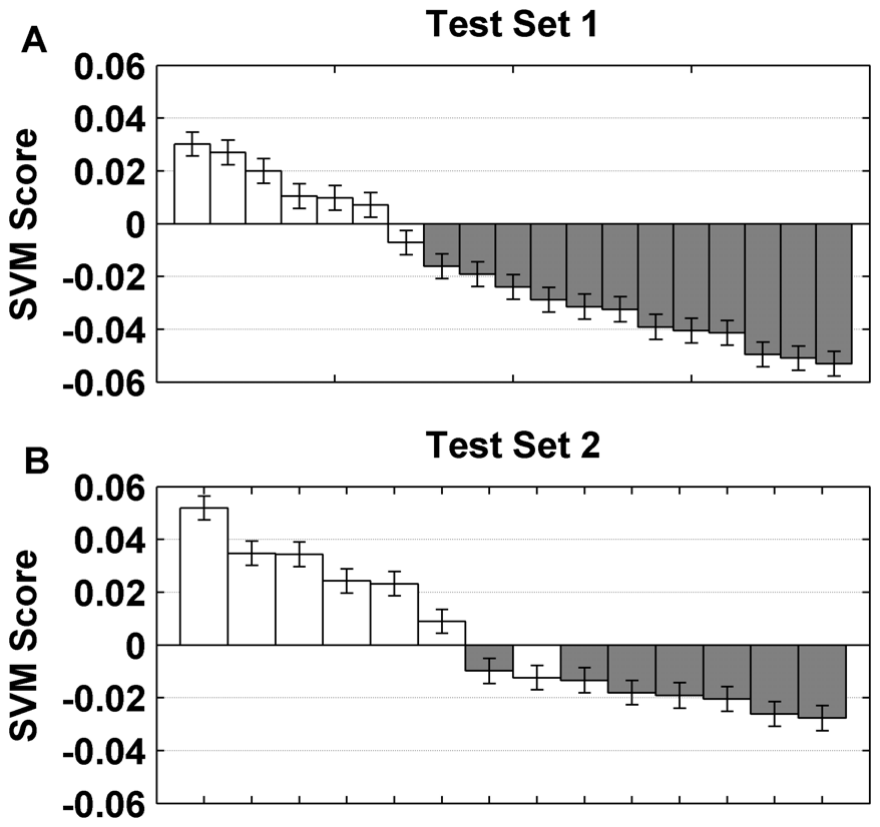
Performance of SVM signature in the testing sets. The classification scores assigned by the SVM based on the 251-gene signature to classify active TB (positive score) and controls (negative scores) in the absence of an HIV infection for A) Test Set 1 classifying TB patients after 2 months of treatment (white) and controls (grey), and B) Test Set 2 classifying TB active patients before treatment (white) and after 12 months of treatment (grey).

To further validate our 251-gene signature and to determine its specificity for the presence of TB, we used five independent, previously published TB datasets available in GEO (Methods, Table 2) [22], [26]. These datasets also allowed us to test the accuracy of the signature in classifying TB infections in the absence of an HIV infection, as none of the patients were infected with HIV. The TB patients in the TS1 dataset (Table 2) most closely resemble those used in our HIV/TB study as all HIV/TB patients were being treated for TB at the time they were sampled. The 251-gene signature yielded a remarkable accuracy of 94.7% on the TS1 dataset, with only one TB patient misclassified as a control, and an area under the ROC curve of 1 (Table 4, Figure 2*A*, Figure S1 *B*). The high accuracy of the signature in this independent test set indicates that our gene signature is TB-specific and can accurately distinguish TB infected patients from uninfected controls even in the absence of HIV even though the TS1 data was collected from whole blood RNA rather than PBMC.

### The 251-gene HIV/TB Signature Correlates with the Presence of a TB Infection

To further test the performance of the 251-gene signature we applied our classifier to Test Set 2 (TS2). TS2 contained the microarray data only from mono-infected TB patients before any treatment and after 12 months of treatment, when they were presumably cured (Table 2). TS2 allowed us to determine whether a TB signature could be detected in patients prior to any treatment with similar accuracy as in TS1. Comparable results to TS1 were observed with the TS2 data (no treatment vs. complete treatment) with an accuracy of 92.9% achieved and only one TB patient being misclassified (Table 4, Figure 2*B*). It should be noted that the TB samples misclassified in TS1 and TS2 were from the same patient. The ability of the signature to correctly classify the pre-treatment TB samples as having TB signifies that the signature is associated with the presence of an active TB infection and not primarily due to the effect of the treatment. This is further supported by the observation that the overall expression of the TB signature in the samples obtained after 12 months of successful treatment resembles the gene profiles of the uninfected controls. A heatmap of the 251 genes in these four groups shows gene expression between the TB patients before and after 2 months of treatment to be quite similar, while expression of these genes in patients 12 months after treatment are more similar to the gene expression levels in the uninfected controls (Figure S2).

The strong correlation of the signature with the presence of a TB infection can be further seen in the principal components analysis plot (Figure S3). The expression of these 251 genes clusters the data across the first principal component in a sequential manner with samples more to less infected clustering from right to left. Samples from TB infected patients prior to treatment cluster furthest to the right, while samples from the early treatment cluster in the middle/right area of the plot closer to the pre-treatment samples. Samples from TB patients taken after 12 months of treatment cluster in the middle/left area of the plot and finally, controls, with and without HIV, cluster to the far left. This progressive relation is also evident in the tracking of the time-series samples for each of the individual patients, represented by the colored lines in the figure. For each of the seven patients the pattern of pre-treatment, 2 months treatment and 12 months treatment samples always move from right to left indicating a change in state from infected to uninfected (Figure S3).

### The 251-gene HIV/TB Signature Distinguishes between Active and Latent TB

We also tested the 251-gene signature’s performance on four additional datasets: TS3 (GSE19439), TS4 (GSE19444), TS5 (GSE19442) and TS6 (GSE40553) to determine whether our signature could also distinguish between untreated active and latent TB infections. Our signature correctly differentiated between active and latent TB infections as well as controls with sensitivities of 69.2% and 76.2% in TS3 and TS4, respectively, less accurately than in the previous analyses (Table 4, Figure S4 *A*–*B*). Most of the misclassified patients were borderline and two patients were within a 0.001 SVM score from the cutoff. Analysis of a larger cohort should allow us to refine our signature so that we can classify these borderline patients more accurately. However, the 76.2% sensitivity achieved in TS4 significantly improves the 61.7% sensitivity previously reported for the 393-transcript signature identified by Berry et al [22]. Moreover, our classifier had a 100% specificity in TS3 and a 97% specificity in TS4, correctly identifying 20 out of the 21 (95.2%) latent TB samples as not having active TB. In addition, the signature correctly identified 18 out of 20 and 25 out of 29 active TB patients (90% and 86.2% sensitivities) and had 90.3% and 92.1% specificities in the TS5 and TS6 South African test sets, respectively (Table 4, Figure S4 *C*–*D*). The accuracy of our signature in distinguishing between active TB and both healthy and latent TB controls is further reinforced by the area under the ROC curve for the four test sets, which ranges between 0.893 and 0.967 (Table 4, Figure S1 *D*–*F*).

### A published 393-gene TB Signature does not Identify TB in Patients Co-infected with HIV

We then tested the performance of the published 393-transcript TB signature [22] on our HIV/TB gene expression data. The 393-gene signature was shown to distinguish patients with active TB from both healthy controls and latent TB patients with 91.7% sensitivity and 96.6% specificity, as reported by Berry et al. [22]. Because of slight differences between Illumina’s HumanHT-12v3 microarrays used for the Berry study and the v4 arrays used for ours, we used the 387 probes from their signature that were common to both arrays to assess whether this gene signature would effectively classify our HIV/TB patients as TB infected. We first applied the k-nearest neighbor (KNN) method similar to that described in [22] using data from the TS3 dataset as a training set. Testing on our data resulted in the correct classification of only 7 out of 21 of our HIV/TB patients (33% sensitivity), indicating that this signature does not perform well on HIV co-infected samples. We also applied the SVM analysis used for our studies as a second approach for testing the performance of the 387 signature. Data from the 387 probes in the TS3 dataset were used to train the model that was then applied to our data. The 387-probe signature was not able to distinguish our two patient classes and classified both mono-infected and co-infected patients as having active TB (0% specificity). Similarly, the 86-gene signature that distinguishes between TB and other inflammatory and infectious diseases [22] could not classify the two groups in our dataset (0% sensitivity) and only had four probes in common with our signature.

## Discussion

There is currently no “gold standard” for TB diagnosis in mono-infected patients or patients co-infected with HIV. While several tests exist for diagnosing TB, the sensitivities for these methods vary largely in the different publications (Table 5). In this study we used SVM-RFE to identify a 251-gene signature that achieved an overall accuracy of 81.4% in distinguishing mono-infected from co-infected HIV/TB patients. The classifier correctly classified 16 out of 21 HIV/TB co-infected patients and 19 out of 22 HIV patients. This signature was similarly accurate when applied to independent data from two large studies of patients mono-infected with TB [22].

**Table 5:** Summary of TB Diagnostic Tests.

Test	Sensitivity	References
Sputum Tests	28.2%	[33]
LAM	28.2–67%	[33], [34]
Xpert MTB/RIF	57–76%	[33], [35]

Sensitivities of currently used TB diagnostic tests.

The overall accuracy of the 251-gene SVM signature was 94.7% when applied to a dataset including healthy controls and TB-infected individuals being treated for 2 months, similar to the treatment of our HIV/TB patients. Remarkably, only one TB patient was misclassified in this study, despite the fact that our data was collected on purified PBMC samples and the mono-infection study was performed on whole blood (Tempus Blood RNA Tubes, Applied Biosystems). These results highlight the robustness of our signature to detect TB both in the presence and absence of an HIV infection and in whole blood samples as well as purified PBMC. We also validated the signature’s ability to distinguish untreated TB samples from samples taken 12 months after treatment when the infection is presumed to be eliminated. While patients only treated for 2 months remain classified as TB-positive, those treated for 12 months are classified as controls. The time course study suggests that our gene signature can assess the efficacy of treatment over time as we find that untreated, 2 months treated and 12 months treated samples from individual patients exhibited a diminished TB signature (score) as a consequence of the treatment. This suggests that the 251-gene signature may be useful in differentiating between patients who respond successfully to treatment (TB eradication) and subjects with poor response who need more aggressive treatment, or with treatment-resistant infections. Larger time-series studies will be needed to evaluate the usefulness of this assessment but Molecular Distance to Health and Temporal Molecular Response scores based on recent data from Bloom et al. [26] support this possibility, as scores for our 251-gene signature are demonstrated to change significantly after two weeks of treatment (Figure S5, Methods and Results S1).

While our training set did not include patients with a latent TB infection, our classifier also accurately distinguished between an active TB infection and a latent infection in the four external datasets tested (TS3-6). Our 251-gene signature correctly identified all 17 patients with latent TB in TS3 (100% sensitivity). In addition, it also classified 20 out of 21 (95.2%), 28 out of 31 (90.3%) and 35 out of 38 (92.1%) patients with a latent TB infection as controls in TS4, TS5 and TS6, respectively.

The recently published 393-gene signature of Berry et al. [22] did not perform well on our HIV-infected samples while our PBMC signature performed accurately on their data. This is likely due to two factors. Berry et al. [22] analyzed gene expression patterns in whole blood specimens collected using Tempus tubes whereas our study used cryopreserved PBMC. Tempus collection tubes capture the neutrophil RNA not captured in the PBMC samples as most of the granulocytes are removed by the PBMC purification.. The other key difference in the two datasets is that the sample population used for the selection of our 251 gene signature was composed entirely of HIV-positive individuals, whereas HIV infection was specifically excluded in Berry’s study. HIV co-infection may obscure the expression of certain genes that would otherwise be related to a TB infection that were detected in the Berry study. Although both signatures clearly demonstrate specificity for the presence of TB, only 16 probes, corresponding to 15 genes, were common to both signatures (TMEM51, APOL6, STAT2, STAT1, LOC653610, GK, DHRS9, TRAFD1, UBE2L6, GBP2, LPCAT2, AK026751, ASPHD2, BRSK1, and FLVCR2). The 393-gene signature [22], has demonstrated excellent sensitivity and specificity in the detection of TB in the absence of HIV while our 251-gene signature could be used for diagnosing the presence of active TB, both in patients with and patients without HIV.

Although 251 genes is a relatively small number for such an analysis, we used Ingenuity (Ingenuity Systems, www.ingenuity.com) to identify pathways and gene functions that might be represented in our 251 gene list. While the list did include genes involved in Immunological Disease (9 genes), Infectious Disease (33 genes) and Inflammatory Disease (7 genes), we did not find any pathways or functions to be significantly enriched after applying the Benjamini–Hochberg procedure for multiple-test correction of p-values (0.05) in the 251 gene list, (Methods and Results S1). In addition to gene expression, we also examined changes in DNA methylation, however, the changes we found were very small and uninformative in our study, likely because they are associated with only a specific cell type in the PBMC mixture of cells. The DNA methylation data are also available in GEO under the accession number GSE50835 (Figure S6; Methods and Results S1).

The size of the cohort used to obtain our 251-gene signature was relatively small, as the collection of the samples analyzed was part of a study that was focused on defining cytokine expression differences between patients infected only with HIV and those co-infected with TB [27]. Thus, we did not enroll TB mono-infected patients or healthy donors. However, the datasets used to validate our TB signature do represent those populations, and the excellent performance of our signature on these large independent datasets strongly supports the validity and robustness of our 251 gene signature. It is possible that this signature could be further reduced with a larger training set, if necessary.

We realize this is only a first step in addressing a difficult area of TB diagnostics and that the major need for such diagnostics are in resource limited settings. We have shown that we could develop a 251 gene signature that improved on the previously described 393 gene signature with the possibility of further improving this signature. While array based assays used to develop this signature remain costly, we and others [31], [32] have successfully moved array developed diagnostics to PCR based platforms which are less costly and less technically demanding. The successful application of our signature to the samples collected in Tempus RNA stabilization tubes as well as to purified PBMC supports the feasibility of collecting samples in field settings and shipping them to a central processing center for analysis. Although much more work is needed these results provide reason for optimism that such diagnostic platforms can be developed.

In conclusion, we report a 251-gene signature that accurately identifies HIV patients that are co-infected with TB. In addition, we show that this signature has broad applicability as it also identifies TB in the absence of an HIV infection. TB infection presents a number of diagnostic challenges, and this is particularly problematic when its association with HIV infection masks its more typical clinical and laboratory presentations. We believe that our signature represents a significant advance, and warrants further testing aimed at determining its impact as a diagnostic tool and/or a means to monitor response to antibacterial treatment.

## Supporting Information

Figure S1 **Receiver operating characteristic curves.** ROC curves depicting the performance of the 251 gene classifier on A) the training set and B–G) the 6 test sets. The area under the curve (AUC) is show in the bottom right corner of each plot.
(TIF)

Figure S2 **Heatmap of the 251 probes in the time series data.** The expression of the 251 probes selected as a classifier through SVM-RFE in TB samples before treatment (left), TB after 2 months of treatment (middle/left), TB samples after 12 months of treatment (middle/right) and controls (right).
(TIF)

Figure S3 **Principal component analysis based on 251 gene signature.** The first principal component shows a progressive pattern from right to left corresponding to TB before treatment (far right) and control (far left) with patients after 12 months of treatment clustering closer to controls. The second principal component reflects the variation due to the presence (bottom) and absence (top) of an HIV infection. Each of the 7 patients is represented by a different color and samples from the different time-points of each patient are connected together.
(TIF)

Figure S4 **Performance of SVM signature in additional testing sets.** Classification scores assigned by the SVM based on the 251 gene signature to classify active TB (positive scores) and controls (negative scores) in A-B) Test Sets 3 and 4 (UK) classifying TB patients (white), latent TB (black) and controls (grey); and C-D) Test Sets 5 and 6 (South Africa) classifying TB patients (white) and latent TB (black). The inserts in each figure show zoomed-in regions of the samples between the dashed vertical lines.
(TIF)

Figure S5 **Changes in expression of the 251 gene associated with treatment.** Significant changes in the expression of the 251 gene signature occur within 2 weeks of treatment as shown by changes in A) Molecular Distance to Health in the South African cohort (median and interquartile range), B) Temporal Molecular Response in the South African cohort (mean and 95% confidence interval) and C) Temporal Molecular Response in the UK cohort (mean and 95% confidence interval). *** = p<0.001, ** = p<0.01 and * = p<0.05.
(TIF)

Figure S6 **Changes in DNA Methylation Levels.** Distribution of genes based on the change in the percent methylation observed between samples from HIV/TB and HIV patients. The 287 genes are those that are both differentially expressed and methylated between the two patient groups. White bars represent genes that are more methylated in HIV/TB than in HIV. Grey bars represent genes that are less methylated in HIV/TB than in HIV.
(TIF)

Table S1 **List of the 251 gene TB-signature identified using SVM-RFE.** Genes are listed according to SVM rank.
(DOCX)

Methods and Results S1 (DOC)
