# Supplementary figures and images for: Identification of a 251 Gene Expression Signature That Can Accurately Detect M. tuberculosis in Patients with and without HIV Co-Infection
Source: PLoS One. 2014 Feb 25;9(2):e89925. doi: 10.1371/journal.pone.0089925 (PMC3934945; doi:10.1371/journal.pone.0089925)

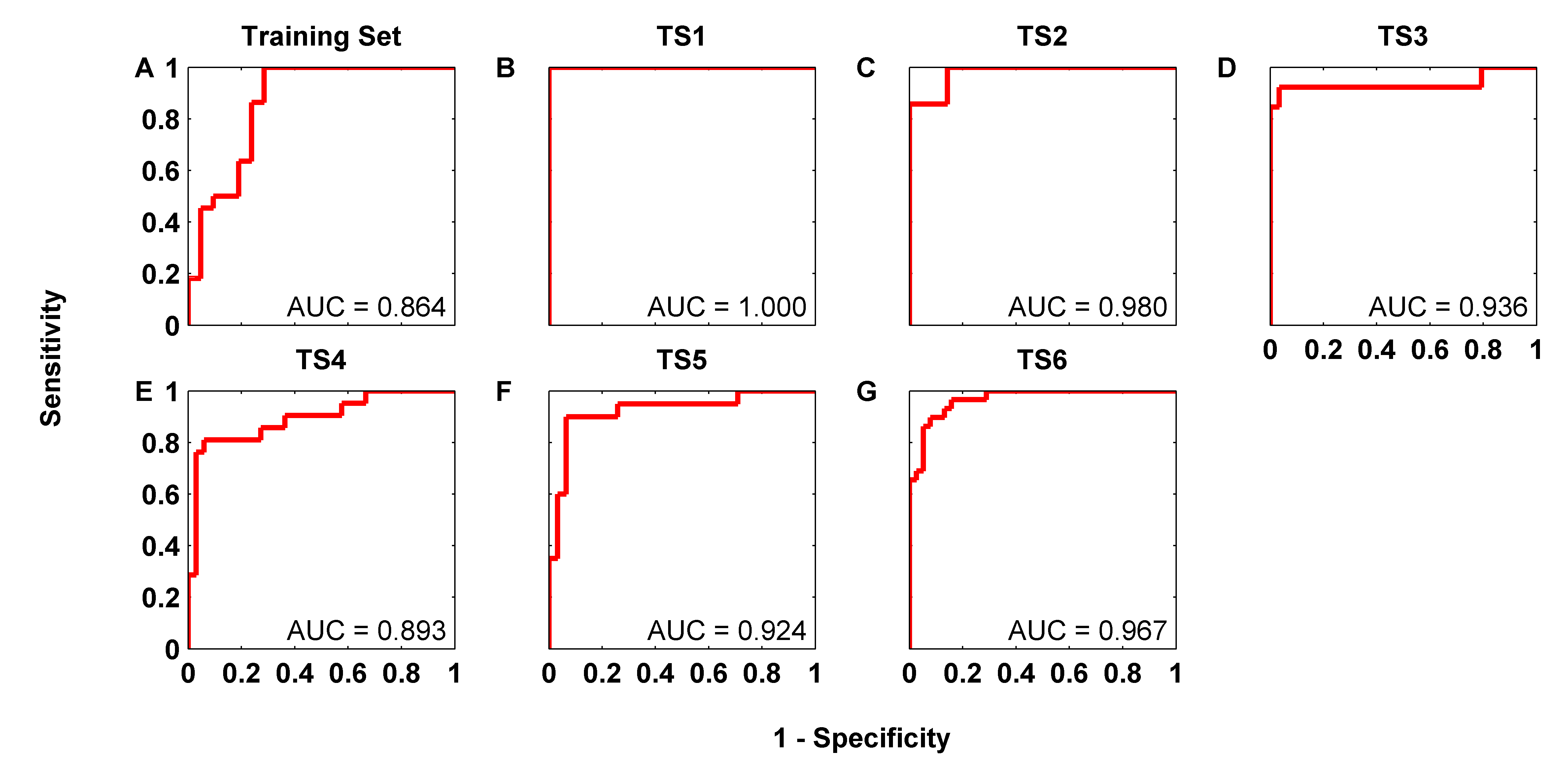

Supplement: Figure S1 — Receiver operating characteristic curves. ROC curves depicting the performance of the 251 gene classifier on A) the training set and B–G) the 6 test sets. The area under the curve (AUC) is show in the bottom right corner of each plot. (TIF) [file pone.0089925.s001.tif]

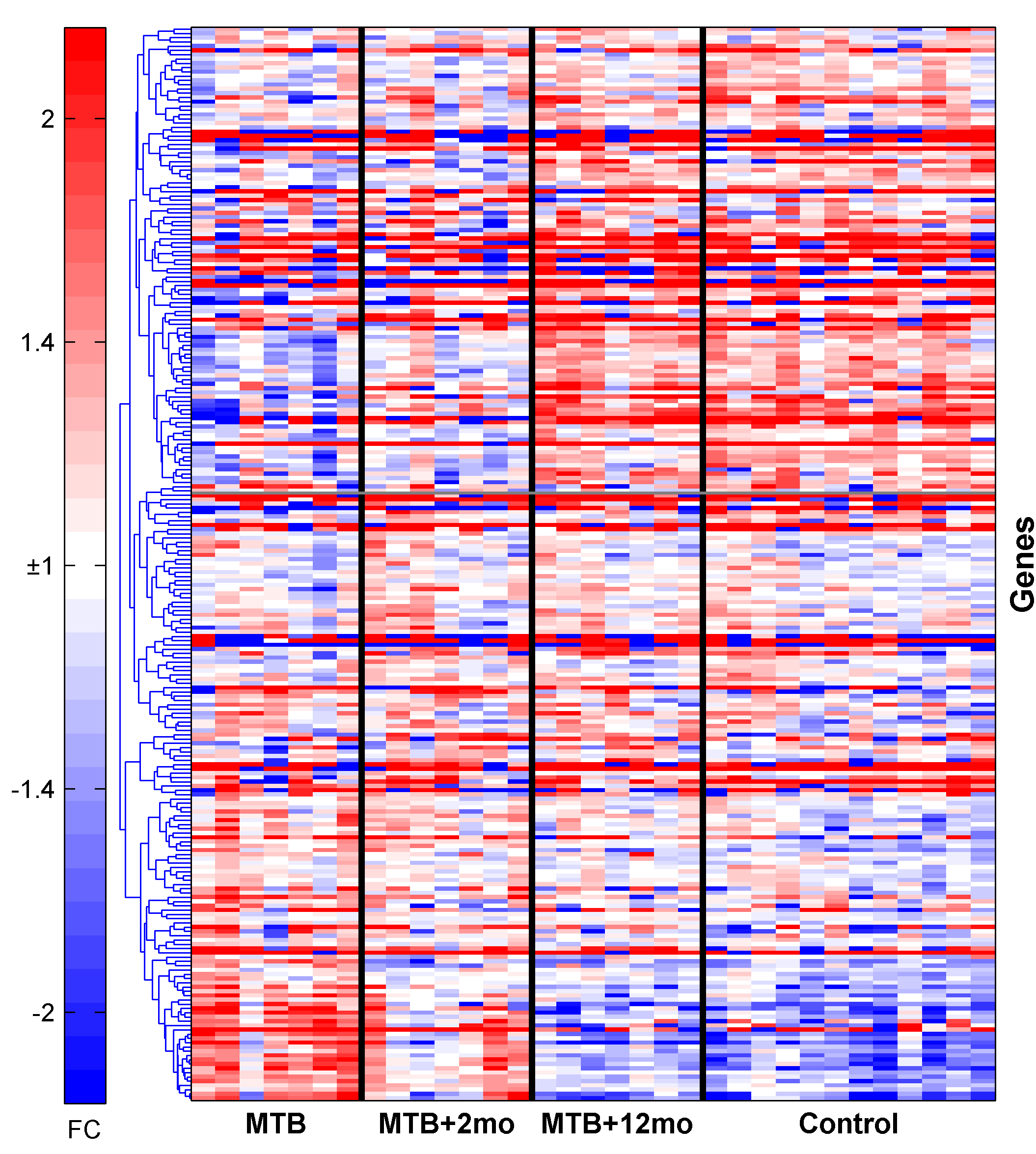

Supplement: Figure S2 — Heatmap of the 251 probes in the time series data. The expression of the 251 probes selected as a classifier through SVM-RFE in TB samples before treatment (left), TB after 2 months of treatment (middle/left), TB samples after 12 months of treatment (middle/right) and controls (right). (TIF) [file pone.0089925.s002.tif]

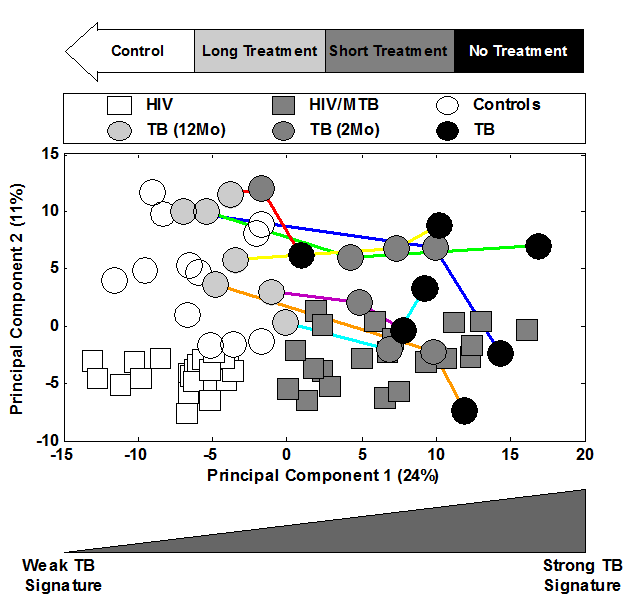

Supplement: Figure S3 — Principal component analysis based on 251 gene signature. The first principal component shows a progressive pattern from right to left corresponding to TB before treatment (far right) and control (far left) with patients after 12 months of treatment clustering closer to controls. The second principal component reflects the variation due to the presence (bottom) and absence (top) of an HIV infection. Each of the 7 patients is represented by a different color and samples from the different time-points of each patient are connected together. (TIF) [file pone.0089925.s003.tif]

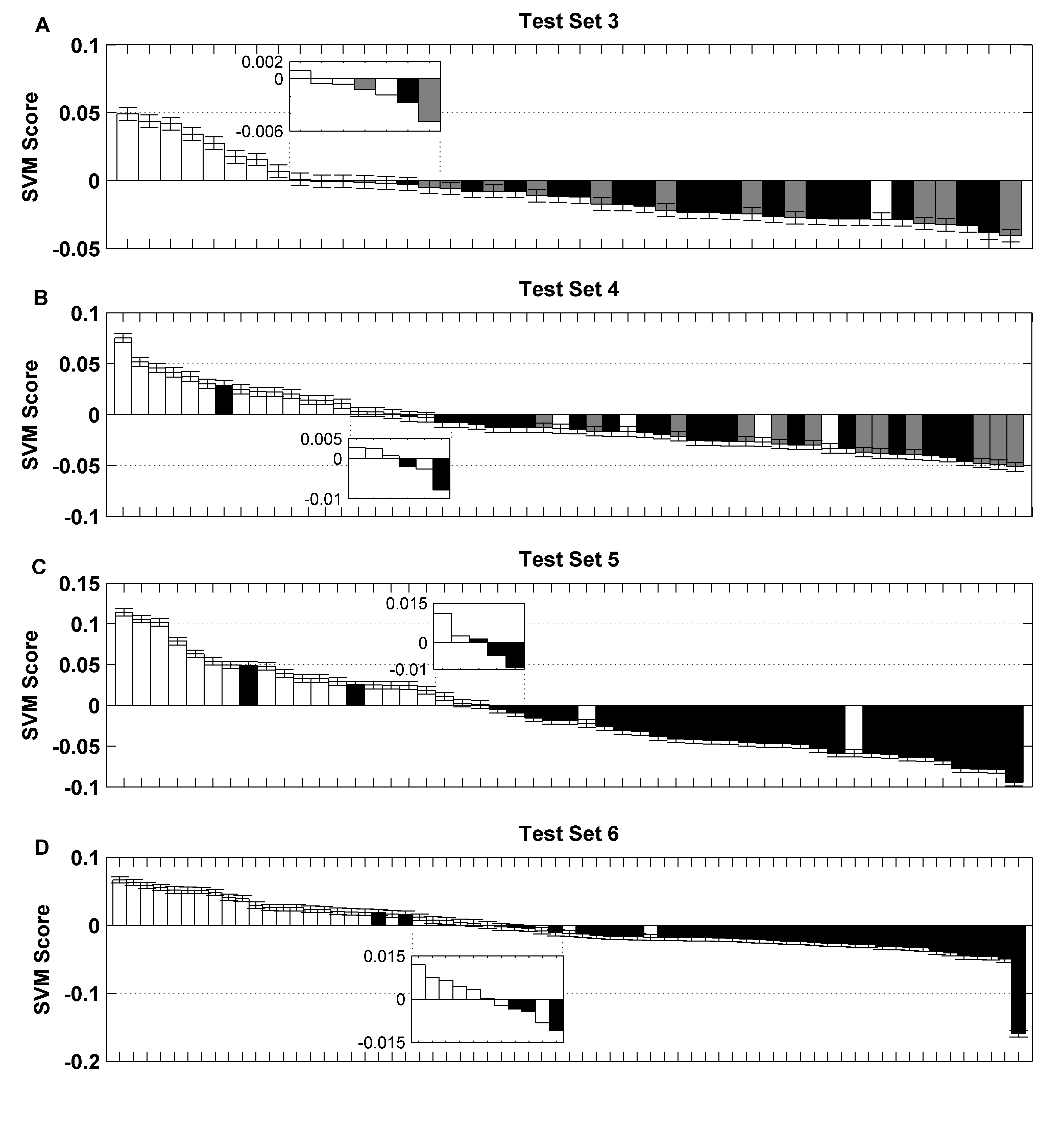

Supplement: Figure S4 — Performance of SVM signature in additional testing sets. Classification scores assigned by the SVM based on the 251 gene signature to classify active TB (positive scores) and controls (negative scores) in A-B) Test Sets 3 and 4 (UK) classifying TB patients (white), latent TB (black) and controls (grey); and C-D) Test Sets 5 and 6 (South Africa) classifying TB patients (white) and latent TB (black). The inserts in each figure show zoomed-in regions of the samples between the dashed vertical lines. (TIF) [file pone.0089925.s004.tif]

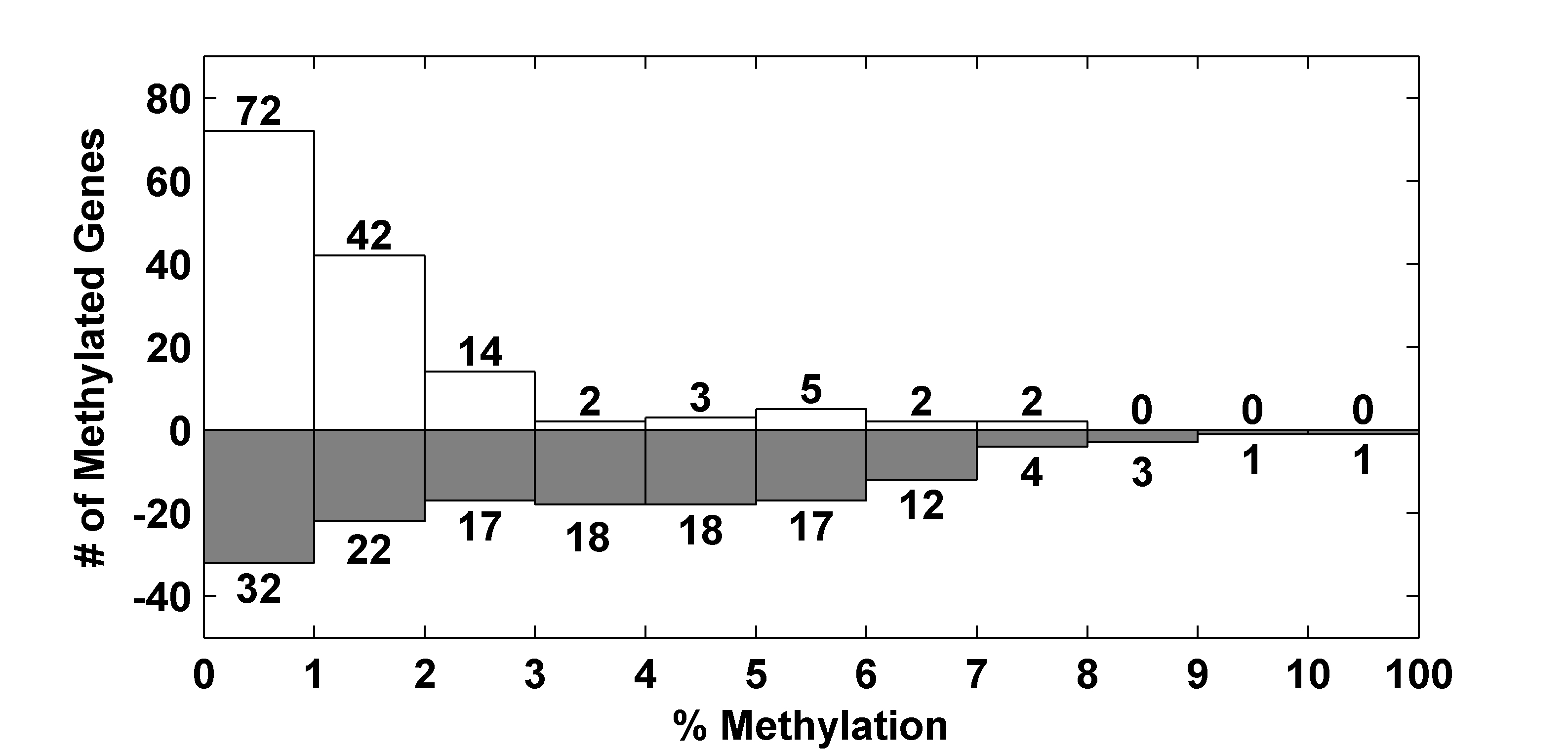

Supplement: Figure S6 — Changes in DNA Methylation Levels. Distribution of genes based on the change in the percent methylation observed between samples from HIV/TB and HIV patients. The 287 genes are those that are both differentially expressed and methylated between the two patient groups. White bars represent genes that are more methylated in HIV/TB than in HIV. Grey bars represent genes that are less methylated in HIV/TB than in HIV. (TIF) [file pone.0089925.s006.tif]
