# Supplementary material for: Identification of a 251 Gene Expression Signature That Can Accurately Detect M. tuberculosis in Patients with and without HIV Co-Infection
Source: PLoS One. 2014 Feb 25;9(2):e89925. doi: 10.1371/journal.pone.0089925 (PMC3934945; doi:10.1371/journal.pone.0089925)
Supplement: Table S1 — List of the 251 gene TB-signature identified using SVM-RFE. Genes are listed according to SVM rank. (DOCX) [file pone.0089925.s007.docx]

**Table S1: List of the 251 gene TB-signature identified using SVM-RFE.** Genes are listed according to SVM rank.

| **Accession** | **Symbol** | **Gene Name** | **Rank** | **Fold Change HIV+TB/HIV** |
| --- | --- | --- | --- | --- |
| NM_021004 | DHRS4 | dehydrogenase/reductase (SDR family) member 4 | 1 | 1.402 |
| NM_001990 | EYA3 | eyes absent homolog 3 (Drosophila) | 2 | 1.172 |
| NM_030629 | CMIP | c-Maf-inducing protein | 3 | 1.248 |
| NM_014233 | UBTF | upstream binding transcription factor, RNA polymerase I | 4 | -1.260 |
| NM_032227 | TMEM164 | transmembrane protein 164 | 5 | 1.492 |
| NM_078487 | CDKN2B | cyclin-dependent kinase inhibitor 2B (p15, inhibits CDK4) | 6 | 1.422 |
| NM_005607 | PTK2 | PTK2 protein tyrosine kinase 2 | 7 | 1.374 |
| XM_939593 | LOC648605 | PREDICTED: similar to Trimethyllysine dioxygenase, mitochondrial precursor | 8 | 1.256 |
| CR621233 | NaN | full-length cDNA clone CS0DI057YA22 of Placenta Cot 25-normalized of (human) | 9 | 1.441 |
| NM_003501 | ACOX3 | acyl-Coenzyme A oxidase 3, pristanoyl | 10 | 1.336 |
| XM_001126647 | MLKL | PREDICTED: mixed lineage kinase domain-like | 11 | 1.454 |
| NM_007100 | ATP5I | ATP synthase, H+ transporting, mitochondrial F0 complex, subunit E | 12 | 1.102 |
| NM_005819 | STX6 | syntaxin 6 | 13 | 1.255 |
| NM_152858 | WTAP | Wilms tumor 1 associated protein | 14 | -1.586 |
| NM_001531 | MR1 | major histocompatibility complex, class I-related | 15 | 1.421 |
| XM_045290 | LOC151579 | PREDICTED: similar to basic leucine zipper and W2 domains 1 | 16 | -1.234 |
| NM_025164 | SIK3 | SIK family kinase 3 | 17 | 1.131 |
| XM_036729 | USP41 | PREDICTED: ubiquitin specific peptidase 41 | 18 | 1.253 |
| NM_145172 | WDR63 | WD repeat domain 63 | 19 | -3.149 |
| NM_023015 | INTS3 | integrator complex subunit 3 | 20 | 1.275 |
| XM_496034 | LOC653717 | PREDICTED: similar to hect domain and RLD 2 | 21 | -1.505 |
| NM_006289 | TLN1 | talin 1 | 22 | 1.248 |
| NM_016533 | NINJ2 | ninjurin 2 | 23 | 1.487 |
| NM_023927 | GRAMD3 | GRAM domain containing 3 | 24 | -1.293 |
| NR_003654 | SCAND2 | SCAN domain containing 2 | 25 | 1.377 |
| NM_031924 | RSPH3 | radial spoke 3 homolog (Chlamydomonas) | 26 | 1.349 |
| NM_194071 | CREB3L2 | cAMP responsive element binding protein 3-like 2 | 27 | 1.196 |
| NM_005484 | PARP2 | poly (ADP-ribose) polymerase family, member 2 | 28 | -1.264 |
| NM_013349 | NENF | neuron derived neurotrophic factor | 29 | 1.222 |
| NM_003820 | TNFRSF14 | tumor necrosis factor receptor superfamily, member 14 (herpesvirus entry mediator) | 30 | 1.186 |
| NM_001044722 | CSNK1G3 | casein kinase 1, gamma 3 | 31 | -1.322 |
| NM_001008218 | AMY1B | amylase, alpha 1B (salivary) | 32 | -1.406 |
| NM_001033925 | TIAL1 | TIA1 cytotoxic granule-associated RNA binding protein-like 1 | 33 | -1.212 |
| NM_145279 | MOBKL2C | MOB1, Mps One Binder kinase activator-like 2C (yeast) | 34 | 1.396 |
| XM_001131304 | LOC728635 | PREDICTED: similar to peroxisomal short-chain alcohol dehydrogenase | 35 | 1.414 |
| XM_208281 | LOC285053 | PREDICTED: similar to ribosomal protein L18a | 36 | -1.227 |
| NM_173654 | C3orf64 | chromosome 3 open reading frame 64 | 37 | -1.259 |
| NM_014709 | USP34 | ubiquitin specific peptidase 34 | 38 | -1.262 |
| AJ420516 | NaN | mRNA full length insert cDNA clone EUROIMAGE 966164 | 39 | -1.455 |
| NM_021136 | RTN1 | reticulon 1 | 40 | 1.577 |
| NM_003758 | EIF3J | eukaryotic translation initiation factor 3, subunit J | 41 | -1.259 |
| NM_014634 | PPM1F | protein phosphatase 1F (PP2C domain containing) | 42 | 1.337 |
| NM_002908 | REL | v-rel reticuloendotheliosis viral oncogene homolog (avian) | 43 | -2.065 |
| NM_032140 | C16orf48 | chromosome 16 open reading frame 48 | 44 | 1.151 |
| NM_005170 | ASCL2 | achaete-scute complex homolog 2 (Drosophila) | 45 | 1.337 |
| NM_005819 | STX6 | syntaxin 6 | 46 | 1.317 |
| NM_001105 | ACVR1 | activin A receptor, type I | 47 | 1.183 |
| NM_003430 | ZNF91 | zinc finger protein 91 | 48 | -1.881 |
| NM_005931 | MICB | MHC class I polypeptide-related sequence B | 49 | 1.343 |
| NM_012228 | MSRB2 | methionine sulfoxide reductase B2 | 50 | 1.676 |
| NM_201437 | TCEA1 | transcription elongation factor A (SII), 1 | 51 | -1.195 |
| NM_001011885 | BTBD1 | BTB (POZ) domain containing 1 | 52 | -1.101 |
| NM_198083 | DHRS4L2 | dehydrogenase/reductase (SDR family) member 4 like 2 | 53 | 1.586 |
| NM_001080499 | LOC402057 | similar to 40S ribosomal protein S17 | 54 | -1.755 |
| NM_134421 | HPCAL1 | hippocalcin-like 1 | 55 | 1.286 |
| NM_023914 | P2RY13 | purinergic receptor P2Y, G-protein coupled, 13 | 56 | 2.152 |
| XM_370865 | LOC388122 | PREDICTED: hypothetical LOC388122 | 57 | -1.483 |
| NM_199189 | MATR3 | matrin 3 | 58 | -1.336 |
| NM_015325 | KIAA0947 | KIAA0947 | 59 | -1.446 |
| NM_030641 | APOL6 | apolipoprotein L, 6 | 60 | 1.531 |
| NM_000167 | GK | glycerol kinase | 61 | 1.803 |
| NM_006120 | HLA-DMA | major histocompatibility complex, class II, DM alpha | 62 | 1.281 |
| NM_004462 | FDFT1 | farnesyl-diphosphate farnesyltransferase 1 | 63 | 1.200 |
| NM_004120 | GBP2 | guanylate binding protein 2, interferon-inducible | 64 | 1.430 |
| NM_006927 | ST3GAL2 | ST3 beta-galactoside alpha-2,3-sialyltransferase 2 | 65 | 1.317 |
| NM_203391 | GK | glycerol kinase | 66 | 1.735 |
| NM_016567 | BCCIP | BRCA2 and CDKN1A interacting protein | 67 | -1.166 |
| NM_032430 | BRSK1 | BR serine/threonine kinase 1 | 68 | 1.996 |
| NM_152649 | MLKL | mixed lineage kinase domain-like | 69 | 1.248 |
| NM_006785 | MALT1 | mucosa associated lymphoid tissue lymphoma translocation gene 1 | 70 | -1.547 |
| NM_014912 | CPEB3 | cytoplasmic polyadenylation element binding protein 3 | 71 | 1.283 |
| XM_944232 | LOC648605 | PREDICTED: similar to Trimethyllysine dioxygenase, mitochondrial precursor | 72 | 1.339 |
| NM_006472 | TXNIP | thioredoxin interacting protein | 73 | 1.203 |
| AI469954 | NaN | tj89a12.x1 Soares_NSF_F8_9W_OT_PA_P_S1 cDNA clone IMAGE:2148670 3 | 74 | 1.328 |
| NM_018022 | TMEM51 | transmembrane protein 51 | 75 | 1.485 |
| NM_001987 | ETV6 | ets variant 6 | 76 | 1.190 |
| NM_001002255 | SUMO4 | SMT3 suppressor of mif two 3 homolog 4 (S. cerevisiae) | 77 | -1.385 |
| NM_178812 | MTDH | metadherin | 78 | -1.220 |
| NM_001002814 | RAB11FIP1 | RAB11 family interacting protein 1 (class I) | 79 | 1.207 |
| NM_014377 | DNAJC2 | DnaJ (Hsp40) homolog, subfamily C, member 2 | 80 | -2.502 |
| XM_374766 | LOC399715 | PREDICTED: FLJ46311 protein | 81 | 1.301 |
| NM_007362 | NCBP2 | nuclear cap binding protein subunit 2, 20kDa | 82 | -1.118 |
| NM_022140 | EPB41L4A | erythrocyte membrane protein band 4.1 like 4A | 83 | 1.334 |
| CN357235 | NaN | 17000532215901 GRN_ES cDNA 5 | 84 | -1.542 |
| XM_496278 | ZNF516 | PREDICTED: zinc finger protein 516 | 85 | 1.351 |
| AK026751 | NaN | cDNA: FLJ23098 fis, clone LNG07440 | 86 | 1.756 |
| XR_018923 | LOC648210 | PREDICTED: similar to Heterogeneous nuclear ribonucleoprotein A1 | 87 | -1.247 |
| NR_002941 | LOC285359 | phosducin-like 3 pseudogene | 88 | -1.483 |
| NM_017791 | FLVCR2 | feline leukemia virus subgroup C cellular receptor family, member 2 | 89 | 1.592 |
| NM_002893 | RBBP7 | retinoblastoma binding protein 7 | 90 | -1.266 |
| NM_002156 | HSPD1 | heat shock 60kDa protein 1 (chaperonin) | 91 | -1.209 |
| NM_003115 | UAP1 | UDP-N-acteylglucosamine pyrophosphorylase 1 | 92 | -1.452 |
| NM_017975 | ZWILCH | Zwilch, kinetochore associated, homolog (Drosophila) | 93 | -1.279 |
| NM_005381 | NCL | nucleolin | 94 | -1.184 |
| NM_006700 | TRAFD1 | TRAF-type zinc finger domain containing 1 | 95 | 1.343 |
| NM_016494 | RNF181 | ring finger protein 181 | 96 | 1.290 |
| NM_007229 | PACSIN2 | protein kinase C and casein kinase substrate in neurons 2 | 97 | 1.371 |
| NM_013232 | PDCD6 | programmed cell death 6 | 98 | 1.122 |
| NM_153689 | FLJ38973 | hypothetical protein FLJ38973 | 99 | -1.689 |
| XM_944889 | WDR43 | PREDICTED: WD repeat domain 43, transcript variant 8 | 100 | -1.251 |
| NM_181524 | PIK3R1 | phosphoinositide-3-kinase, regulatory subunit 1 (alpha) | 101 | -1.619 |
| NM_012478 | WBP2 | WW domain binding protein 2 | 102 | 1.178 |
| NM_018067 | RPRC1 | arginine/proline rich coiled-coil 1 | 103 | 1.205 |
| NM_024579 | C1orf54 | chromosome 1 open reading frame 54 | 104 | 1.356 |
| NM_021184 | C6orf47 | chromosome 6 open reading frame 47 | 105 | 1.279 |
| CR743148 | NaN | CR743148 NCI_CGAP_GC4 cDNA clone IMAGp971L0563 ; IMAGE:1550800 5 | 106 | -1.306 |
| NM_005740 | DNAL4 | dynein, axonemal, light chain 4 | 107 | 1.259 |
| NM_024874 | KIAA0319L | KIAA0319-like | 108 | 1.142 |
| NM_006713 | SUB1 | SUB1 homolog (S. cerevisiae) | 109 | -1.210 |
| NM_016145 | C19orf56 | chromosome 19 open reading frame 56 | 110 | 1.148 |
| NM_153188 | TNPO1 | transportin 1 | 111 | -1.245 |
| NM_000402 | G6PD | glucose-6-phosphate dehydrogenase | 112 | 1.309 |
| NM_001099776 | LOC91431 | prematurely terminated mRNA decay factor-like | 113 | -1.354 |
| XM_937107 | LOC648057 | PREDICTED: similar to Guanine nucleotide-binding protein G(s), alpha subunit (Adenylate cyclase-stimulating G alpha protein) (G-alpha-8) | 114 | -1.448 |
| NR_002448 | SNORD36A | small nucleolar RNA, C/D box 36A | 115 | -1.336 |
| NM_079421 | CDKN2D | cyclin-dependent kinase inhibitor 2D (p19, inhibits CDK4) | 116 | 1.205 |
| NM_006769 | LMO4 | LIM domain only 4 | 117 | 1.143 |
| NM_022091 | ASCC3 | activating signal cointegrator 1 complex subunit 3 | 118 | -1.533 |
| NM_020192 | C7orf36 | chromosome 7 open reading frame 36 | 119 | -1.426 |
| NM_005797 | MPZL2 | myelin protein zero-like 2 | 120 | 2.195 |
| NM_017455 | NPTN | neuroplastin | 121 | 1.186 |
| NM_170695 | TGIF1 | TGFB-induced factor homeobox 1 | 122 | -1.358 |
| NM_173469 | UBE2Q2 | ubiquitin-conjugating enzyme E2Q family member 2 | 123 | -1.236 |
| BG911604 | NaN | 602812728F1 NCI_CGAP_Brn67 cDNA clone IMAGE:4944798 5 | 124 | 1.257 |
| NM_004071 | CLK1 | CDC-like kinase 1 | 125 | -1.699 |
| NM_001080394 | KIAA0146 | KIAA0146 | 126 | 1.302 |
| NM_015435 | RNF19A | ring finger protein 19A | 127 | -1.347 |
| NM_006156 | NEDD8 | neural precursor cell expressed, developmentally down-regulated 8 | 128 | 1.116 |
| XR_000900 | LOC390354 | PREDICTED: similar to ribosomal protein L18a; 60S ribosomal protein L18a, transcript variant 36 | 129 | -1.217 |
| NM_139312 | YME1L1 | YME1-like 1 (S. cerevisiae) | 130 | -1.156 |
| NM_017839 | LPCAT2 | lysophosphatidylcholine acyltransferase 2 | 131 | 2.375 |
| BM728567 | NaN | UI-E-EJ0-aiu-f-01-0-UI.r1 UI-E-EJ0 cDNA clone UI-E-EJ0-aiu-f-01-0-UI 5 | 132 | 1.279 |
| NM_031411 | PCDHA1 | protocadherin alpha 1 | 133 | 1.317 |
| NM_020347 | LZTFL1 | leucine zipper transcription factor-like 1 | 134 | -1.520 |
| NM_002688 | SEPT5 | septin 5 | 135 | 1.739 |
| NM_017785 | CCDC99 | coiled-coil domain containing 99 | 136 | -1.550 |
| XM_937850 | LOC285176 | PREDICTED: similar to ribosomal protein L10 | 137 | -1.364 |
| NM_019600 | KIAA1370 | KIAA1370 | 138 | -1.324 |
| NM_020409 | MRPL47 | mitochondrial ribosomal protein L47 | 139 | -2.035 |
| NM_153045 | C9orf91 | chromosome 9 open reading frame 91 | 140 | 1.291 |
| NM_017769 | KIAA1333 | KIAA1333 | 141 | -1.610 |
| NM_014612 | FAM120A | family with sequence similarity 120A | 142 | 1.101 |
| XM_929628 | LOC644380 | PREDICTED: similar to High mobility group protein 1-like 10 (HMG-1L10) | 143 | -1.606 |
| NM_021019 | MYL6 | myosin, light chain 6, alkali, smooth muscle and non-muscle | 144 | 1.164 |
| NM_014670 | BZW1 | basic leucine zipper and W2 domains 1 | 145 | -1.521 |
| NM_020437 | ASPHD2 | aspartate beta-hydroxylase domain containing 2 | 146 | 1.459 |
| NM_001040876 | ABCE1 | ATP-binding cassette, sub-family E | 147 | -1.242 |
| NM_032154 | PCGF6 | polycomb group ring finger 6 | 148 | -1.516 |
| NM_022117 | TSPYL2 | TSPY-like 2 | 149 | -1.455 |
| NM_005437 | NCOA4 | nuclear receptor coactivator 4 | 150 | 1.305 |
| NM_022754 | SFXN1 | sideroflexin 1 | 151 | -1.316 |
| NM_002198 | IRF1 | interferon regulatory factor 1 | 152 | 1.292 |
| NM_001042532 | COASY | Coenzyme A synthase | 153 | 1.188 |
| NM_016151 | TAOK2 | TAO kinase 2 | 154 | 1.374 |
| NM_001031677 | RAB24 | RAB24, member RAS oncogene family | 155 | 1.425 |
| NM_153207 | AEBP2 | AE binding protein 2 | 156 | -1.210 |
| NM_004156 | PPP2CB | protein phosphatase 2 (formerly 2A), catalytic subunit, beta isoform | 157 | 1.181 |
| NM_002118 | HLA-DMB | major histocompatibility complex, class II, DM beta | 158 | 1.384 |
| NM_004719 | SFRS2IP | splicing factor, arginine/serine-rich 2, interacting protein | 159 | -1.326 |
| AK026966 | NaN | cDNA: FLJ23313 fis, clone HEP11919 | 160 | 2.224 |
| NM_006447 | USP16 | ubiquitin specific peptidase 16 | 161 | -1.323 |
| NM_002267 | KPNA3 | karyopherin alpha 3 (importin alpha 4) | 162 | -1.201 |
| NM_005907 | MAN1A1 | mannosidase, alpha, class 1A, member 1 | 163 | -1.224 |
| NR_003263 | SDHAP3 | succinate dehydrogenase complex, subunit A, flavoprotein pseudogene 3 | 164 | -1.630 |
| NM_017902 | HIF1AN | hypoxia inducible factor 1, alpha subunit inhibitor | 165 | 1.273 |
| NM_002635 | SLC25A3 | solute carrier family 25 (mitochondrial carrier; phosphate carrier), member 3 | 166 | -1.084 |
| NM_005389 | PCMT1 | protein-L-isoaspartate (D-aspartate) O-methyltransferase | 167 | 1.250 |
| NM_014670 | BZW1 | basic leucine zipper and W2 domains 1 | 168 | -1.475 |
| NM_003431 | ZNF124 | zinc finger protein 124 | 169 | -1.367 |
| NM_153363 | ZNF679 | zinc finger protein 679 | 170 | -1.848 |
| NM_130772 | S100Z | S100 calcium binding protein Z | 171 | 1.422 |
| NM_005419 | STAT2 | signal transducer and activator of transcription 2, 113kDa | 172 | 1.418 |
| NM_000377 | WAS | Wiskott-Aldrich syndrome (eczema-thrombocytopenia) | 173 | 1.169 |
| NM_021004 | DHRS4 | dehydrogenase/reductase (SDR family) member 4 | 174 | 1.364 |
| NM_001656 | TRIM23 | tripartite motif-containing 23 | 175 | -1.329 |
| AA177072 | NaN | nc02h04.s1 NCI_CGAP_Pr3 cDNA clone IMAGE:280 | 176 | -1.248 |
| NM_000243 | MEFV | Mediterranean fever | 177 | 2.322 |
| NM_015361 | R3HDM1 | R3H domain containing 1 | 178 | -1.148 |
| NM_005771 | DHRS9 | dehydrogenase/reductase (SDR family) member 9 | 179 | 1.674 |
| XM_928387 | LOC653610 | PREDICTED: similar to Histone H2A.o (H2A/o) (H2A.2) (H2a-615) | 180 | 1.351 |
| NM_012079 | DGAT1 | diacylglycerol O-acyltransferase homolog 1 (mouse) | 181 | 1.206 |
| NM_058246 | DNAJB6 | DnaJ (Hsp40) homolog, subfamily B, member 6 | 182 | 1.333 |
| NM_003017 | SFRS3 | splicing factor, arginine/serine-rich 3 | 183 | -1.266 |
| NM_152991 | EED | embryonic ectoderm development | 184 | -1.511 |
| AI732736 | NaN | zw18g10.x5 Soares ovary tumor NbHOT cDNA clone IMAGE:769698 3 similar to TR:O54850 O54850 ENDONUCLEASE/ REVERSE TRANSCRIPTASE | 185 | 1.408 |
| NM_004876 | ZNF254 | zinc finger protein 254 | 186 | -1.222 |
| NM_153697 | ANKRD44 | ankyrin repeat domain 44 | 187 | -1.382 |
| NM_001259 | CDK6 | cyclin-dependent kinase 6 | 188 | -1.312 |
| NM_001031623 | ZNF451 | zinc finger protein 451 | 189 | -1.590 |
| NM_001018160 | NAE1 | NEDD8 activating enzyme E1 subunit 1 | 190 | -1.460 |
| NM_002912 | REV3L | REV3-like, catalytic subunit of DNA polymerase zeta (yeast) | 191 | -1.543 |
| NM_000885 | ITGA4 | integrin, alpha 4 (antigen CD49D, alpha 4 subunit of VLA-4 receptor) | 192 | -1.286 |
| NM_005499 | UBA2 | ubiquitin-like modifier activating enzyme 2 | 193 | -1.265 |
| NM_016134 | PGCP | plasma glutamate carboxypeptidase | 194 | 1.411 |
| NM_016072 | GOLT1B | golgi transport 1 homolog B (S. cerevisiae) | 195 | -1.294 |
| NM_001018089 | NARG2 | NMDA receptor regulated 2 | 196 | -1.556 |
| NM_199418 | PRCP | prolylcarboxypeptidase (angiotensinase C) | 197 | 1.257 |
| NM_017858 | TIPIN | TIMELESS interacting protein | 198 | -2.035 |
| NR_003028 | SNORA25 | small nucleolar RNA, H/ACA box 25 | 199 | -1.538 |
| NM_001225 | CASP4 | caspase 4, apoptosis-related cysteine peptidase | 200 | 1.254 |
| NM_015885 | PCF11 | PCF11, cleavage and polyadenylation factor subunit, homolog (S. cerevisiae) | 201 | -1.212 |
| XM_945579 | LOC649555 | PREDICTED: similar to eukaryotic translation initiation factor 4E | 202 | -1.343 |
| NM_000367 | TPMT | thiopurine S-methyltransferase | 203 | 1.182 |
| NM_006267 | RANBP2 | RAN binding protein 2 | 204 | -1.683 |
| NM_018393 | TCP11L1 | t-complex 11 (mouse)-like 1 | 205 | 1.223 |
| NM_005381 | NCL | nucleolin | 206 | -1.279 |
| XM_942687 | LOC654189 | PREDICTED: similar to heterogeneous nuclear ribonucleoprotein A3 | 207 | -1.375 |
| NM_018471 | ZC3H15 | zinc finger CCCH-type containing 15 | 208 | -1.181 |
| NM_000402 | G6PD | glucose-6-phosphate dehydrogenase | 209 | 1.309 |
| NM_017567 | NAGK | N-acetylglucosamine kinase | 210 | 1.480 |
| NM_182760 | SUMF1 | sulfatase modifying factor 1 | 211 | 1.566 |
| NM_199141 | CARM1 | coactivator-associated arginine methyltransferase 1 | 212 | 1.194 |
| NM_002704 | PPBP | pro-platelet basic protein (chemokine (C-X-C motif) ligand 7) | 213 | 1.859 |
| NM_014929 | FASTKD2 | FAST kinase domains 2 | 214 | -1.258 |
| NM_022153 | C10orf54 | chromosome 10 open reading frame 54 | 215 | 1.367 |
| NM_006203 | PDE4D | phosphodiesterase 4D, cAMP-specific (phosphodiesterase E3 dunce homolog, Drosophila) | 216 | -1.609 |
| CA421847 | NaN | UI-H-FG0-bcw-h-18-0-UI.s1 NCI_CGAP_EN1_2 cDNA clone UI-H-FG0-bcw-h-18-0-UI 3 | 217 | 1.257 |
| NM_033360 | KRAS | v-Ki-ras2 Kirsten rat sarcoma viral oncogene homolog | 218 | -1.259 |
| NM_000487 | ARSA | arylsulfatase A | 219 | 1.269 |
| NM_145644 | MRPL35 | mitochondrial ribosomal protein L35 | 220 | 1.264 |
| NM_005737 | ARL4C | ADP-ribosylation factor-like 4C | 221 | -1.547 |
| NM_005904 | SMAD7 | SMAD family member 7 | 222 | -1.590 |
| NR_003032 | SNORA32 | small nucleolar RNA, H/ACA box 32 | 223 | -1.947 |
| NM_005190 | CCNC | cyclin C | 224 | -1.176 |
| NM_004223 | UBE2L6 | ubiquitin-conjugating enzyme E2L 6 | 225 | 1.354 |
| NM_001013663 | C2orf79 | chromosome 2 open reading frame 79 | 226 | 1.230 |
| XM_938703 | TMLHE | PREDICTED: trimethyllysine hydroxylase, epsilon | 227 | 1.358 |
| XM_931232 | LOC90120 | PREDICTED: hypothetical gene supported by AK023162 | 228 | 1.196 |
| NM_006785 | MALT1 | mucosa associated lymphoid tissue lymphoma translocation gene 1 | 229 | -1.308 |
| NM_207585 | IFNAR2 | interferon (alpha, beta and omega) receptor 2 | 230 | 1.312 |
| NM_019044 | FLJ10996 | hypothetical protein FLJ10996 | 231 | 1.144 |
| XM_937113 | LOC647436 | PREDICTED: similar to ribosomal protein L5 | 232 | -1.549 |
| NM_016283 | TAF9 | TAF9 RNA polymerase II, TATA box binding protein (TBP)-associated factor, 32kDa | 233 | -1.811 |
| NM_006985 | NPIP | nuclear pore complex interacting protein | 234 | -1.268 |
| NM_139313 | YME1L1 | YME1-like 1 (S. cerevisiae) | 235 | -1.155 |
| NM_003797 | EED | embryonic ectoderm development | 236 | -1.329 |
| NM_020787 | ZNF624 | zinc finger protein 624 | 237 | -1.408 |
| NM_022662 | ANAPC1 | anaphase promoting complex subunit 1 | 238 | -1.154 |
| NM_024804 | ZNF669 | zinc finger protein 669 | 239 | -1.297 |
| NM_139266 | STAT1 | signal transducer and activator of transcription 1, 91kDa | 240 | 1.435 |
| NM_030918 | SNX27 | sorting nexin family member 27 | 241 | 1.474 |
| NM_130807 | MOBKL2A | MOB1, Mps One Binder kinase activator-like 2A (yeast) | 242 | 1.249 |
| NM_001017963 | HSP90AA1 | heat shock protein 90kDa alpha (cytosolic), class A member 1 | 243 | -1.163 |
| NM_032186 | ZNF644 | zinc finger protein 644 | 244 | -1.444 |
| NM_014878 | KIAA0020 | KIAA0020 | 245 | -1.222 |
| NR_003277 | LOC728643 | heterogeneous nuclear ribonucleoprotein A1 pseudogene | 246 | -1.270 |
| NM_001071775 | LOC440145 | similar to RIKEN cDNA 2410129H14 | 247 | -1.493 |
| NM_024775 | GEMIN6 | gem (nuclear organelle) associated protein 6 | 248 | 1.398 |
| NM_030637 | DDHD1 | DDHD domain containing 1 | 249 | -1.400 |
| NM_014739 | BCLAF1 | BCL2-associated transcription factor 1 | 250 | -1.375 |
| NM_007097 | CLTB | clathrin, light chain (Lcb) | 251 | 1.165 |
