# Supplementary material for: Identification of a 251 Gene Expression Signature That Can Accurately Detect M. tuberculosis in Patients with and without HIV Co-Infection
Source: PLoS One. 2014 Feb 25;9(2):e89925. doi: 10.1371/journal.pone.0089925 (PMC3934945; doi:10.1371/journal.pone.0089925)
Supplement: Methods and Results S1 — (DOC) [file pone.0089925.s008.doc]

***Methods and Results S1:***

***Methods:***

**Active and Latent TB Diagnosis**

In the South African cohort used in our study, active TB was confirmed using Ziehl-Neelson staining on sputum smears. Subjects with negative sputum smear and subjects with prior TB diagnoses also underwent mycobacterial culture. Chest X-rays were also performed on patients.

For Test Sets 1-6 (UK and Sotuh Africa), patients with active TB were confirmed by laboratory isolation of *M. tuberculosis* on mycobacterial culture of a respiratory specimen (either sputum or bronchoalveolar lavage fluid). Latent TB diagnosis for Test Sets 1-4 (UK ) was based on a positive TST and a positive IGRA result. For Test Sets 5 and 6, IGRA positivity alone was used to confirm the latent TB diagnosis, irrespective of TST result. Plain chest radiographs were obtained for all patients recruited in London.

**DNA Methylation Microarrays.**

Total DNA was isolated from PBMCs using the modified protocol of Sigma Aldrich Tri-reagent (cat #T9424). Isolated DNA at 500ng was bisulfite converted using the Zymo Research EZ DNA Methylation Kit (cat # D5001) for methylation analysis. The DNA was hybridized to the Illumina Infinium 27k Human Methylation array. Genome Studio was used to obtain beta values that correspond to the level of methylation of a probe ranging from 0 for completely unmethylated to 1 for completely methylated. Raw methylation data were quantile normalized. The threshold cutoff was set three standard deviations above the mean expression of the negative probes. For each probe, if at least one sample had an undetectable signal from both channels, the probe was removed from the data. DNA methylation data is available in GEO under the accession number GSE50835.

**Differential Gene Expression and Functional Analysis**.

Supervised analysis was performed as described to identify probes that were significantly different between HIV and HIV/TB patients in the gene expression and DNA methylation data. A t-test (p<0.05) was used and *P*-values were corrected for multiple testing using the false discovery rate [[1](#_ENREF_1)]. Biological functions and pathways significantly enriched with the probes that were differentially expressed between mono-infected and co-infected patients were identified using IPA (Ingenuity Systems, www.ingenuity.com). Significance was determined using the Benjamini and Hochberg multiple-test corrected *P*-value at a cutoff of 0.05.

**Response to Treatment**.

To examine the changes in expression of our gene signature gene in response to treatment, we analyzed temporal data from the South African and UK cohorts published in GSE40553 [[2](#_ENREF_2)]. Due to discrepancies between the annotation provided in the publication and the sample description of the data published in GEO for the UK dataset, we assumed that samples labeled as treated for 1, 2, 3, 4, and 5 months in GEO corresponded to no treatment, 2 weeks, 2 months, 4 months and 6 months of treatment, respectively. Molecular Distance to Health (MDTH) and Temporal Molecular Response (TMR) were calculated for each time point, as previously described by Bloom et al. [[2](#_ENREF_2)]. *P*-values for the pairwise comparisons between the different time points were determined using a linear mixed-effects model.

***Results:***

In addition to the identification of a predictive gene expression signature, we also examined the overall changes in gene expression and DNA methylation associated with the TB infection in HIV co-infected patients. Using a t-test, our analysis identified a total of 4,684 probes that were expressed at significantly different levels between mono-infected HIV patients and co-infected HIV/TB patients (p < 0.05, FDR = 8%). Meanwhile, we identified 2,786 probes for 2,590 unique genes that were differentially methylated (p < 0.05, FDR = 38%) between the two groups. Of these, 287 genes exhibited changes in DNA methylation that were inversely correlated with the observed increases and decreases in gene expression, although the changes in gene-specific methylation levels between the two groups were relatively small (Supplementary Figure 6). Since this is likely due to the fact that changes are only associated with a specific cell type within the PBMC cell population, we further analyzed the 287 genes using the Immune Response in Silico (IRIS) database [[3](#_ENREF_3)] to determine whether the genes were primarily expressed in a specific immune cell type. We found that 21 out of 287 genes were specific to myeloid cells, (a significant result; p = 3.12x10^-7^, enrichment = 3.88), suggesting an involvement, at least in part, of cells of the myeloid lineage.

While functional analysis of our 251-gene signature did not reveal any pathways or biological functions that were significantly enriched, IPA analysis of all differentially expressed genes (p < 0.05) identified Infectious Disease, Respiratory Disease, Immunological Disease and Inflammatory Disease as the biological functions that were highly significantly associated with those genes. This list was also significantly enriched for probes associated with important signaling pathways including Glucocorticoid Receptor Signaling, Integrin Signaling, IL-4 Signaling, and CTLA4 Signaling in Cytotoxic T Lymphocytes. Additional enriched pathways associated with specific immune cell subsets included Fcγ Receptor-mediated Phagocytosis in Macrophages and Monocytes, Dendritic Cell Maturation, and Production of Nitric Oxide and Reactive Oxygen Species in Macrophages, suggesting a more prominent involvement of these cellular subsets in disease.

In order to assess whether there are changes in the expression levels of the 251 genes in our signature that are induced by treatment, we calculated the MDTH and TMR (Supplementary Figure 2). Similar to the signatures reported in [[2](#_ENREF_2)], the most significant change in expression, as determined by both the MDTH and TMR, is seen after 2 weeks of treatment. The MDTH continues to progressively decrease till 6 months (Supplementary Figure 5*A*), while the TMR stabilizes and remains at a constant level after 2 months (Supplementary Figure 5*B*-*C*).

***References:***

1. Storey JD, Tibshirani R (2003) Statistical methods for identifying differentially expressed genes in DNA microarrays. Methods in molecular biology 224: 149-157.
2. Bloom CI, Graham CM, Berry MP, Wilkinson KA, Oni T, et al. (2012) Detectable changes in the blood transcriptome are present after two weeks of antituberculosis therapy. PloS one 7: e46191.
3. Abbas AR, Baldwin D, Ma Y, Ouyang W, Gurney A, et al. (2005) Immune response in silico (IRIS): immune-specific genes identified from a compendium of microarray expression data. Genes Immun 6: 319-331.
